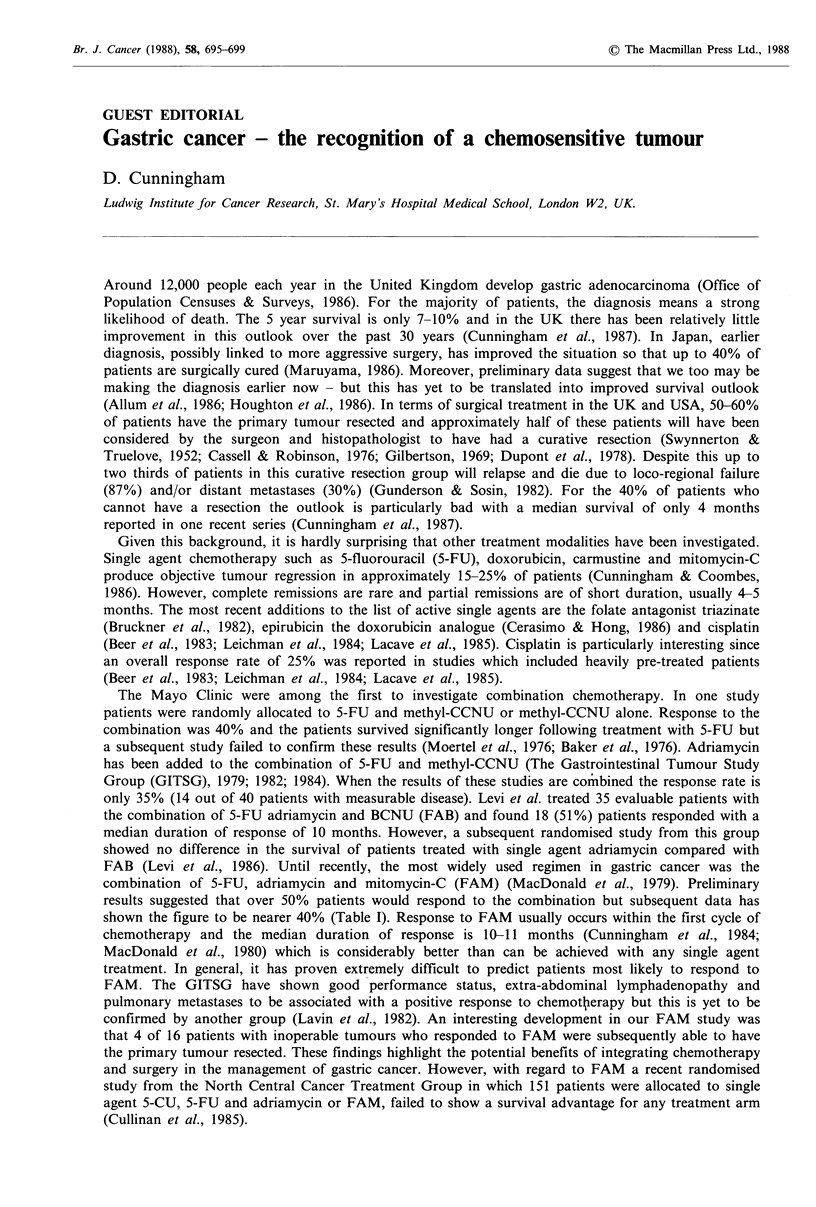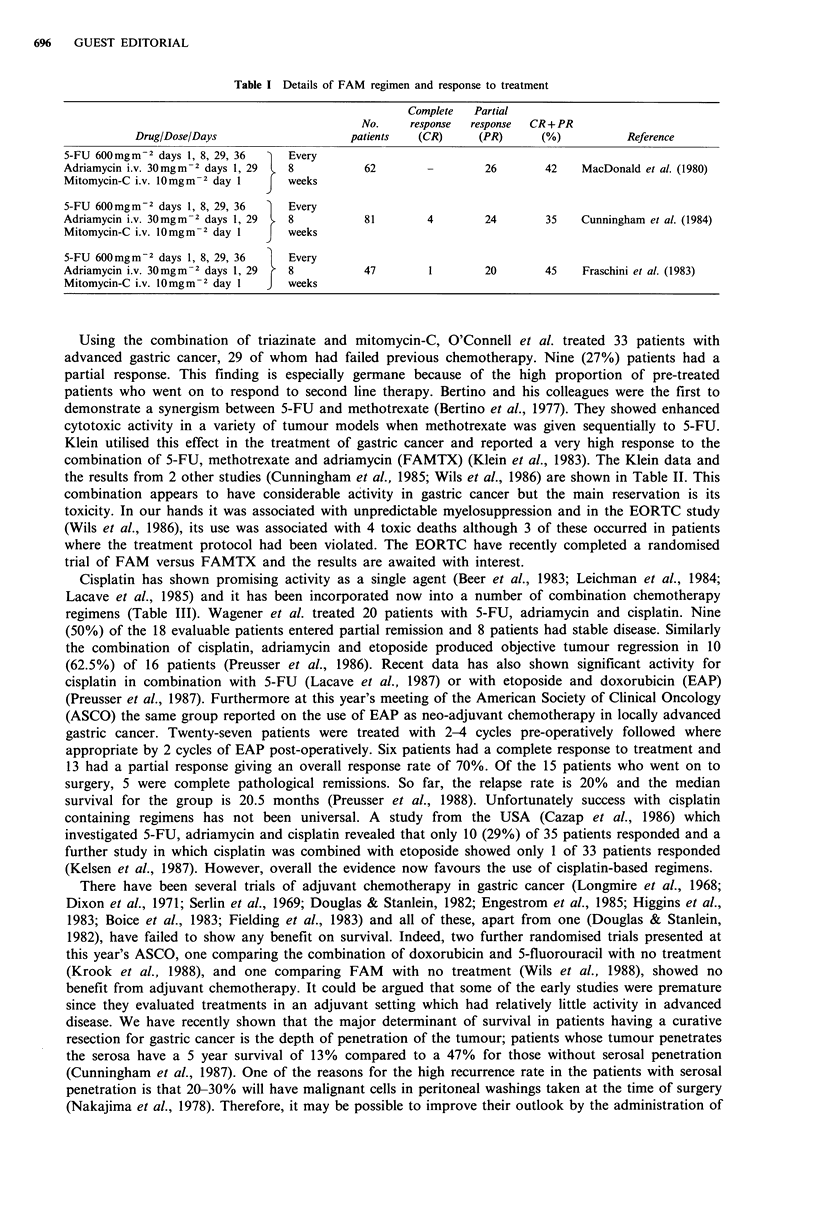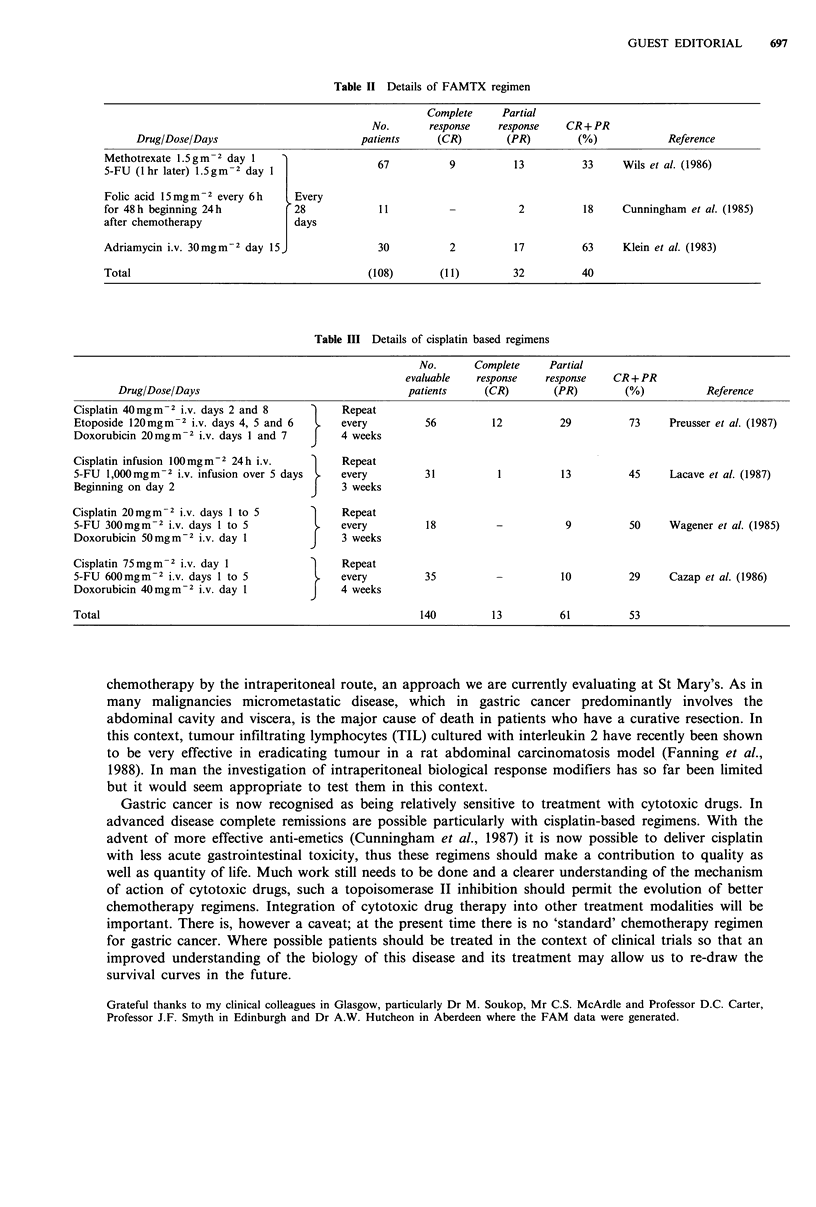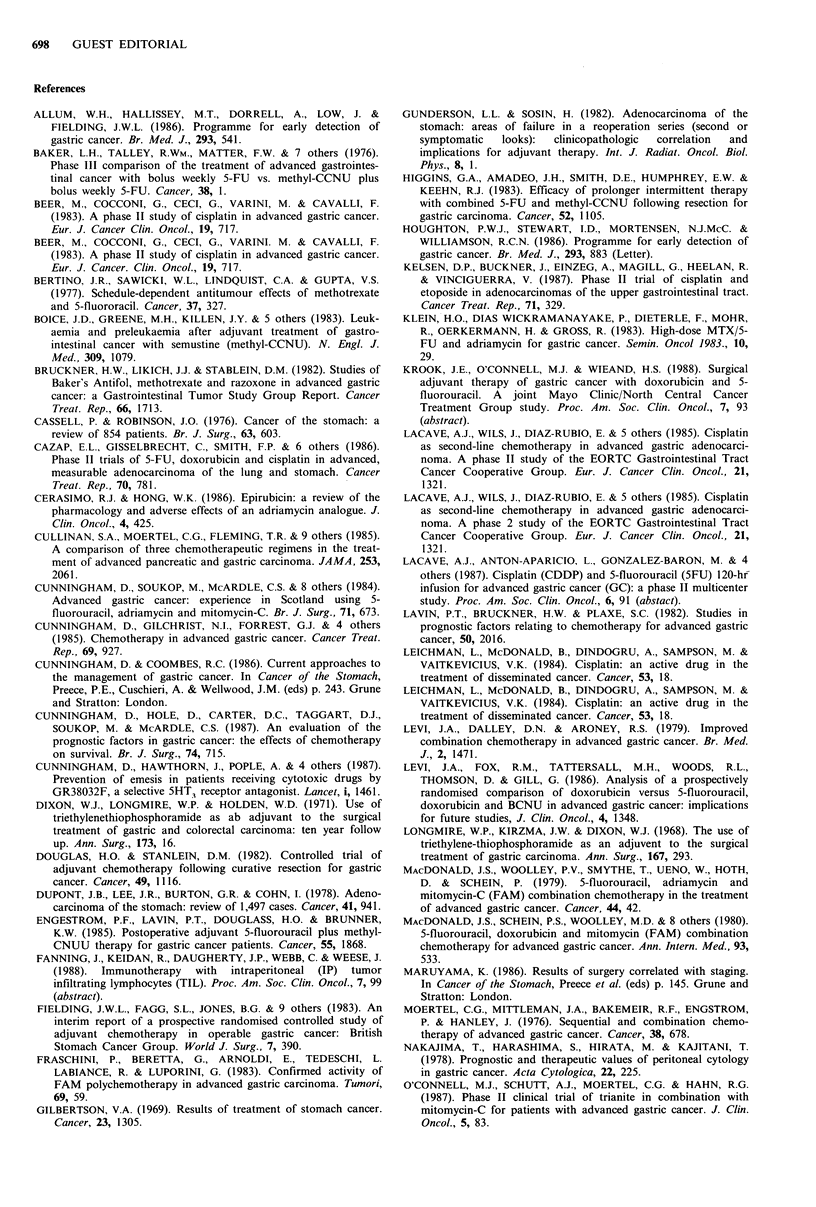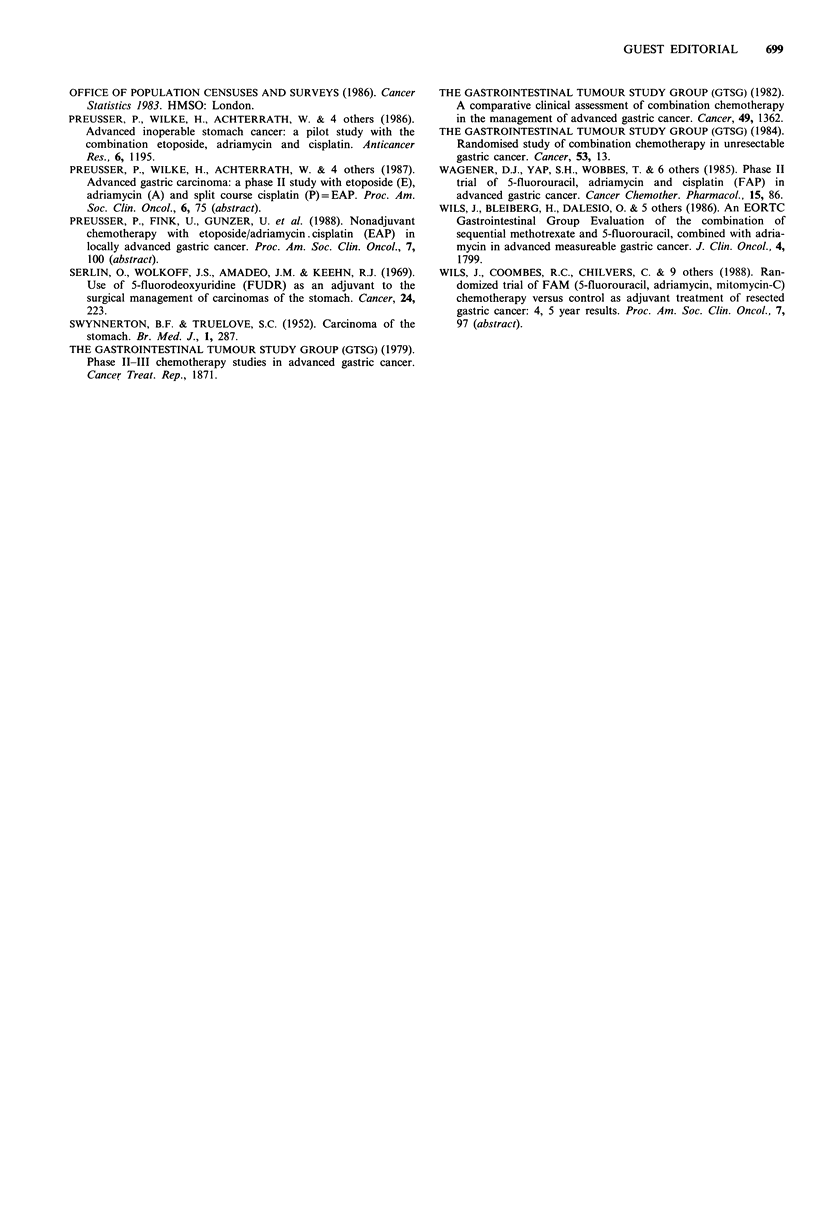# Gastric cancer--the recognition of a chemosensitive tumour.

**DOI:** 10.1038/bjc.1988.292

**Published:** 1988-12

**Authors:** D. Cunningham

**Affiliations:** Ludwig Institute for Cancer Research, St. Mary's Hospital Medical School, London, UK.


Br. J. Cancer (1988), 58, 695–699 © The Macmillan Press Ltd., 1988

GUEST EDITORIAL

### Gastric cancer – the recognition of a chemosensitive tumour

D. Cunningham

*Ludwig Institute for Cancer Research, St. Mary's Hospital Medical School, London W2, UK.*

Around 12,000 people each year in the United Kingdom develop gastric adenocarcinoma (Office of Population Censuses & Surveys, 1986). For the majority of patients, the diagnosis means a strong likelihood of death. The 5 year survival is only 7–10% and in the UK there has been relatively little improvement in this outlook over the past 30 years (Cunningham *et al.*, 1987). In Japan, earlier diagnosis, possibly linked to more aggressive surgery, has improved the situation so that up to 40% of patients are surgically cured (Maruyama, 1986). Moreover, preliminary data suggest that we too may be making the diagnosis earlier now – but this has yet to be translated into improved survival outlook (Allum *et al.*, 1986; Houghton *et al.*, 1986). In terms of surgical treatment in the UK and USA, 50–60% of patients have the primary tumour resected and approximately half of these patients will have been considered by the surgeon and histopathologist to have had a curative resection (Swynnerton & Truelove, 1952; Cassell & Robinson, 1976; Gilbertson, 1969; Dupont *et al.*, 1978). Despite this up to two thirds of patients in this curative resection group will relapse and die due to loco-regional failure (87%) and/or distant metastases (30%) (Gunderson & Sosin, 1982). For the 40% of patients who cannot have a resection the outlook is particularly bad with a median survival of only 4 months reported in one recent series (Cunningham *et al.*, 1987).

Given this background, it is hardly surprising that other treatment modalities have been investigated. Single agent chemotherapy such as 5-fluorouracil (5-FU), doxorubicin, carmustine and mitomycin-C produce objective tumour regression in approximately 15–25% of patients (Cunningham & Coombes, 1986). However, complete remissions are rare and partial remissions are of short duration, usually 4–5 months. The most recent additions to the list of active single agents are the folate antagonist triazinate (Bruckner *et al.*, 1982), epirubicin the doxorubicin analogue (Cerasimo & Hong, 1986) and cisplatin (Beer *et al.*, 1983; Leichman *et al.*, 1984; Lacave *et al.*, 1985). Cisplatin is particularly interesting since an overall response rate of 25% was reported in studies which included heavily pre-treated patients (Beer *et al.*, 1983; Leichman *et al.*, 1984; Lacave *et al.*, 1985).

The Mayo Clinic were among the first to investigate combination chemotherapy. In one study patients were randomly allocated to 5-FU and methyl-CCNU or methyl-CCNU alone. Response to the combination was 40% and the patients survived significantly longer following treatment with 5-FU but a subsequent study failed to confirm these results (Moertel *et al.*, 1976; Baker *et al.*, 1976). Adriamycin has been added to the combination of 5-FU and methyl-CCNU (The Gastrointestinal Tumour Study Group (GITSG), 1979; 1982; 1984). When the results of these studies are combined the response rate is only 35% (14 out of 40 patients with measurable disease). Levi *et al.* treated 35 evaluable patients with the combination of 5-FU adriamycin and BCNU (FAB) and found 18 (51%) patients responded with a median duration of response of 10 months. However, a subsequent randomised study from this group showed no difference in the survival of patients treated with single agent adriamycin compared with FAB (Levi *et al.*, 1986). Until recently, the most widely used regimen in gastric cancer was the combination of 5-FU, adriamycin and mitomycin-C (FAM) (MacDonald *et al.*, 1979). Preliminary results suggested that over 50% patients would respond to the combination but subsequent data has shown the figure to be nearer 40% (Table I). Response to FAM usually occurs within the first cycle of chemotherapy and the median duration of response is 10–11 months (Cunningham *et al.*, 1984; MacDonald *et al.*, 1980) which is considerably better than can be achieved with any single agent treatment. In general, it has proven extremely difficult to predict patients most likely to respond to FAM. The GITSG have shown good performance status, extra-abdominal lymphadenopathy and pulmonary metastases to be associated with a positive response to chemotherapy but this is yet to be confirmed by another group (Lavin *et al.*, 1982). An interesting development in our FAM study was that 4 of 16 patients with inoperable tumours who responded to FAM were subsequently able to have the primary tumour resected. These findings highlight the potential benefits of integrating chemotherapy and surgery in the management of gastric cancer. However, with regard to FAM a recent randomised study from the North Central Cancer Treatment Group in which 151 patients were allocated to single agent 5-CU, 5-FU and adriamycin or FAM, failed to show a survival advantage for any treatment arm (Cullinan *et al.*, 1985).

**Table I** Details of FAM regimen and response to treatment

| *Drug/Dose/Days* | | *No. patients* | *Complete response (CR)* | *Partial response (PR)* | *CR+PR (%)* | *Reference* |
|---|---|---|---|---|---|---|
| 5-FU 600 mg m$^{-2}$ days 1, 8, 29, 36<br>Adriamycin i.v. 30 mg m$^{-2}$ days 1, 29<br>Mitomycin-C i.v. 10 mg m$^{-2}$ day 1 | Every 8 weeks | 62 | – | 26 | 42 | MacDonald *et al.* (1980) |
| 5-FU 600 mg m$^{-2}$ days 1, 8, 29, 36<br>Adriamycin i.v. 30 mg m$^{-2}$ days 1, 29<br>Mitomycin-C i.v. 10 mg m$^{-2}$ day 1 | Every 8 weeks | 81 | 4 | 24 | 35 | Cunningham *et al.* (1984) |
| 5-FU 600 mg m$^{-2}$ days 1, 8, 29, 36<br>Adriamycin i.v. 30 mg m$^{-2}$ days 1, 29<br>Mitomycin-C i.v. 10 mg m$^{-2}$ day 1 | Every 8 weeks | 47 | 1 | 20 | 45 | Fraschini *et al.* (1983) |

Using the combination of triazinate and mitomycin-C, O'Connell *et al.* treated 33 patients with advanced gastric cancer, 29 of whom had failed previous chemotherapy. Nine (27%) patients had a partial response. This finding is especially germane because of the high proportion of pre-treated patients who went on to respond to second line therapy. Bertino and his colleagues were the first to demonstrate a synergism between 5-FU and methotrexate (Bertino *et al.*, 1977). They showed enhanced cytotoxic activity in a variety of tumour models when methotrexate was given sequentially to 5-FU. Klein utilised this effect in the treatment of gastric cancer and reported a very high response to the combination of 5-FU, methotrexate and adriamycin (FAMTX) (Klein *et al.*, 1983). The Klein data and the results from 2 other studies (Cunningham *et al.*, 1985; Wils *et al.*, 1986) are shown in Table II. This combination appears to have considerable activity in gastric cancer but the main reservation is its toxicity. In our hands it was associated with unpredictable myelosuppression and in the EORTC study (Wils *et al.*, 1986), its use was associated with 4 toxic deaths although 3 of these occurred in patients where the treatment protocol had been violated. The EORTC have recently completed a randomised trial of FAM versus FAMTX and the results are awaited with interest.

Cisplatin has shown promising activity as a single agent (Beer *et al.*, 1983; Leichman *et al.*, 1984; Lacave *et al.*, 1985) and it has been incorporated now into a number of combination chemotherapy regimens (Table III). Wagener *et al.* treated 20 patients with 5-FU, adriamycin and cisplatin. Nine (50%) of the 18 evaluable patients entered partial remission and 8 patients had stable disease. Similarly the combination of cisplatin, adriamycin and etoposide produced objective tumour regression in 10 (62.5%) of 16 patients (Preusser *et al.*, 1986). Recent data has also shown significant activity for cisplatin in combination with 5-FU (Lacave *et al.*, 1987) or with etoposide and doxorubicin (EAP) (Preusser *et al.*, 1987). Furthermore at this year's meeting of the American Society of Clinical Oncology (ASCO) the same group reported on the use of EAP as neo-adjuvant chemotherapy in locally advanced gastric cancer. Twenty-seven patients were treated with 2–4 cycles pre-operatively followed where appropriate by 2 cycles of EAP post-operatively. Six patients had a complete response to treatment and 13 had a partial response giving an overall response rate of 70%. Of the 15 patients who went on to surgery, 5 were complete pathological remissions. So far, the relapse rate is 20% and the median survival for the group is 20.5 months (Preusser *et al.*, 1988). Unfortunately success with cisplatin containing regimens has not been universal. A study from the USA (Cazap *et al.*, 1986) which investigated 5-FU, adriamycin and cisplatin revealed that only 10 (29%) of 35 patients responded and a further study in which cisplatin was combined with etoposide showed only 1 of 33 patients responded (Kelsen *et al.*, 1987). However, overall the evidence now favours the use of cisplatin-based regimens.

There have been several trials of adjuvant chemotherapy in gastric cancer (Longmire *et al.*, 1968; Dixon *et al.*, 1971; Serlin *et al.*, 1969; Douglas & Stanlein, 1982; Engestrom *et al.*, 1985; Higgins *et al.*, 1983; Boice *et al.*, 1983; Fielding *et al.*, 1983) and all of these, apart from one (Douglas & Stanlein, 1982), have failed to show any benefit on survival. Indeed, two further randomised trials presented at this year's ASCO, one comparing the combination of doxorubicin and 5-fluorouracil with no treatment (Krook *et al.*, 1988), and one comparing FAM with no treatment (Wils *et al.*, 1988), showed no benefit from adjuvant chemotherapy. It could be argued that some of the early studies were premature since they evaluated treatments in an adjuvant setting which had relatively little activity in advanced disease. We have recently shown that the major determinant of survival in patients having a curative resection for gastric cancer is the depth of penetration of the tumour; patients whose tumour penetrates the serosa have a 5 year survival of 13% compared to a 47% for those without serosal penetration (Cunningham *et al.*, 1987). One of the reasons for the high recurrence rate in the patients with serosal penetration is that 20–30% will have malignant cells in peritoneal washings taken at the time of surgery (Nakajima *et al.*, 1978). Therefore, it may be possible to improve their outlook by the administration of

**Table II** Details of FAMTX regimen

| *Drug/Dose/Days* | | *No. patients* | *Complete response (CR)* | *Partial response (PR)* | *CR+PR (%)* | *Reference* |
|---|---|---|---|---|---|---|
| Methotrexate 1.5 gm$^{-2}$ day 1<br>5-FU (1 hr later) 1.5 gm$^{-2}$ day 1 | Every 28 days | 67 | 9 | 13 | 33 | Wils *et al.* (1986) |
| Folic acid 15 mgm$^{-2}$ every 6 h for 48 h beginning 24 h after chemotherapy | | 11 | – | 2 | 18 | Cunningham *et al.* (1985) |
| Adriamycin i.v. 30 mgm$^{-2}$ day 15 | | 30 | 2 | 17 | 63 | Klein *et al.* (1983) |
| Total | | (108) | (11) | 32 | 40 | |

**Table III** Details of cisplatin based regimens

| *Drug/Dose/Days* | | *No. evaluable patients* | *Complete response (CR)* | *Partial response (PR)* | *CR+PR (%)* | *Reference* |
|---|---|---|---|---|---|---|
| Cisplatin 40 mgm$^{-2}$ i.v. days 2 and 8<br>Etoposide 120 mgm$^{-2}$ i.v. days 4, 5 and 6<br>Doxorubicin 20 mgm$^{-2}$ i.v. days 1 and 7 | Repeat every 4 weeks | 56 | 12 | 29 | 73 | Preusser *et al.* (1987) |
| Cisplatin infusion 100 mgm$^{-2}$ 24 h i.v.<br>5-FU 1,000 mgm$^{-2}$ i.v. infusion over 5 days<br>Beginning on day 2 | Repeat every 3 weeks | 31 | 1 | 13 | 45 | Lacave *et al.* (1987) |
| Cisplatin 20 mgm$^{-2}$ i.v. days 1 to 5<br>5-FU 300 mgm$^{-2}$ i.v. days 1 to 5<br>Doxorubicin 50 mgm$^{-2}$ i.v. day 1 | Repeat every 3 weeks | 18 | – | 9 | 50 | Wagener *et al.* (1985) |
| Cisplatin 75 mgm$^{-2}$ i.v. day 1<br>5-FU 600 mgm$^{-2}$ i.v. days 1 to 5<br>Doxorubicin 40 mgm$^{-2}$ i.v. day 1 | Repeat every 4 weeks | 35 | – | 10 | 29 | Cazap *et al.* (1986) |
| Total | | 140 | 13 | 61 | 53 | |

chemotherapy by the intraperitoneal route, an approach we are currently evaluating at St Mary's. As in many malignancies micrometastatic disease, which in gastric cancer predominantly involves the abdominal cavity and viscera, is the major cause of death in patients who have a curative resection. In this context, tumour infiltrating lymphocytes (TIL) cultured with interleukin 2 have recently been shown to be very effective in eradicating tumour in a rat abdominal carcinomatosis model (Fanning *et al.*, 1988). In man the investigation of intraperitoneal biological response modifiers has so far been limited but it would seem appropriate to test them in this context.

Gastric cancer is now recognised as being relatively sensitive to treatment with cytotoxic drugs. In advanced disease complete remissions are possible particularly with cisplatin-based regimens. With the advent of more effective anti-emetics (Cunningham *et al.*, 1987) it is now possible to deliver cisplatin with less acute gastrointestinal toxicity, thus these regimens should make a contribution to quality as well as quantity of life. Much work still needs to be done and a clearer understanding of the mechanism of action of cytotoxic drugs, such a topoisomerase II inhibition should permit the evolution of better chemotherapy regimens. Integration of cytotoxic drug therapy into other treatment modalities will be important. There is, however a caveat; at the present time there is no 'standard' chemotherapy regimen for gastric cancer. Where possible patients should be treated in the context of clinical trials so that an improved understanding of the biology of this disease and its treatment may allow us to re-draw the survival curves in the future.

Grateful thanks to my clinical colleagues in Glasgow, particularly Dr M. Soukop, Mr C.S. McArdle and Professor D.C. Carter, Professor J.F. Smyth in Edinburgh and Dr A.W. Hutcheon in Aberdeen where the FAM data were generated.

**References**

ALLUM, W.H., HALLISSEY, M.T., DORRELL, A., LOW, J. & FIELDING, J.W.L. (1986). Programme for early detection of gastric cancer. *Br. Med. J.*, **293**, 541.

BAKER, L.H., TALLEY, R.WM., MATTER, F.W. & 7 others (1976). Phase III comparison of the treatment of advanced gastrointestinal cancer with bolus weekly 5-FU vs. methyl-CCNU plus bolus weekly 5-FU. *Cancer*, **38**, 1.

BEER, M., COCCONI, G., CECI, G., VARINI, M. & CAVALLI, F. (1983). A phase II study of cisplatin in advanced gastric cancer. *Eur. J. Cancer Clin. Oncol.*, **19**, 717.

BEER, M., COCCONI, G., CECI, G., VARINI. M. & CAVALLI, F. (1983). A phase II study of cisplatin in advanced gastric cancer. *Eur. J. Cancer. Clin. Oncol.*, **19**, 717.

BERTINO, J.R., SAWICKI, W.L., LINDQUIST, C.A. & GUPTA, V.S. (1977). Schedule-dependent antitumour effects of methotrexate and 5-fluororacil. *Cancer*, **37**, 327.

BOICE, J.D., GREENE, M.H., KILLEN, J.Y. & 5 others (1983). Leukaemia and preleukaemia after adjuvant treatment of gastrointestinal cancer with semustine (methyl-CCNU). *N. Engl. J. Med.*, **309**, 1079.

BRUCKNER, H.W., LIKICH, J.J. & STABLEIN, D.M. (1982). Studies of Baker's Antifol, methotrexate and razoxone in advanced gastric cancer: a Gastrointestinal Tumor Study Group Report. *Cancer Treat. Rep.*, **66**, 1713.

CASSELL, P. & ROBINSON, J.O. (1976). Cancer of the stomach: a review of 854 patients. *Br. J. Surg.*, **63**, 603.

CAZAP, E.L., GISSELBRECHT, C., SMITH, F.P. & 6 others (1986). Phase II trials of 5-FU, doxorubicin and cisplatin in advanced, measurable adenocarcinoma of the lung and stomach. *Cancer Treat. Rep.*, **70**, 781.

CERASIMO, R.J. & HONG, W.K. (1986). Epirubicin: a review of the pharmacology and adverse effects of an adriamycin analogue. *J. Clin. Oncol.*, **4**, 425.

CULLINAN, S.A., MOERTEL, C.G., FLEMING, T.R. & 9 others (1985). A comparison of three chemotherapeutic regimens in the treatment of advanced pancreatic and gastric carcinoma. *JAMA*, **253**, 2061.

CUNNINGHAM, D., SOUKOP, M., McARDLE, C.S. & 8 others (1984). Advanced gastric cancer: experience in Scotland using 5-fluorouracil, adriamycin and mitomycin-C. *Br. J. Surg.*, **71**, 673.

CUNNINGHAM, D., GILCHRIST, N.I., FORREST, G.J. & 4 others (1985). Chemotherapy in advanced gastric cancer. *Cancer Treat. Rep.*, **69**, 927.

CUNNINGHAM, D. & COOMBES, R.C. (1986). Current approaches to the management of gastric cancer. In *Cancer of the Stomach*, Preece, P.E., Cuschieri, A. & Wellwood, J.M. (eds) p. 243. Grune and Stratton: London.

CUNNINGHAM, D., HOLE, D., CARTER, D.C., TAGGART, D.J., SOUKOP, M. & McARDLE, C.S. (1987). An evaluation of the prognostic factors in gastric cancer: the effects of chemotherapy on survival. *Br. J. Surg.*, **74**, 715.

CUNNINGHAM, D., HAWTHORN, J., POPLE, A. & 4 others (1987). Prevention of emesis in patients receiving cytotoxic drugs by GR38032F, a selective $5HT_3$ receptor antagonist. *Lancet*, **i**, 1461.

DIXON, W.J., LONGMIRE, W.P. & HOLDEN, W.D. (1971). Use of triethylenethiophosphoramide as ab adjuvant to the surgical treatment of gastric and colorectal carcinoma: ten year follow up. *Ann. Surg.*, **173**, 16.

DOUGLAS, H.O. & STANLEIN, D.M. (1982). Controlled trial of adjuvant chemotherapy following curative resection for gastric cancer. *Cancer*, **49**, 1116.

DUPONT, J.B., LEE, J.R., BURTON, G.R. & COHN, I. (1978). Adenocarcinoma of the stomach: review of 1,497 cases. *Cancer*, **41**, 941.

ENGESTROM, P.F., LAVIN, P.T., DOUGLASS, H.O. & BRUNNER, K.W. (1985). Postoperative adjuvant 5-fluorouracil plus methyl-CNUU therapy for gastric cancer patients. *Cancer*, **55**, 1868.

FANNING, J., KEIDAN, R., DAUGHERTY, J.P., WEBB, C. & WEESE, J. (1988). Immunotherapy with intraperitoneal (IP) tumor infiltrating lymphocytes (TIL). *Proc. Am. Soc. Clin. Oncol.*, **7**, 99 (*abstract*).

FIELDING, J.W.L., FAGG, S.L., JONES, B.G. & 9 others (1983). An interim report of a prospective randomised controlled study of adjuvant chemotherapy in operable gastric cancer: British Stomach Cancer Group. *World J. Surg.*, **7**, 390.

FRASCHINI, P., BERETTA, G., ARNOLDI, E., TEDESCHI, L. LABIANCE, R. & LUPORINI, G. (1983). Confirmed activity of FAM polychemotherapy in advanced gastric carcinoma. *Tumori*, **69**, 59.

GILBERTSON, V.A. (1969). Results of treatment of stomach cancer. *Cancer*, **23**, 1305.

GUNDERSON, L.L. & SOSIN, H. (1982). Adenocarcinoma of the stomach: areas of failure in a reoperation series (second or symptomatic looks): clinicopathologic correlation and implications for adjuvant therapy. *Int. J. Radiat. Oncol. Biol. Phys.*, **8**, 1.

HIGGINS, G.A., AMADEO, J.H., SMITH, D.E., HUMPHREY, E.W. & KEEHN, R.J. (1983). Efficacy of prolonger intermittent therapy with combined 5-FU and methyl-CCNU following resection for gastric carcinoma. *Cancer*, **52**, 1105.

HOUGHTON, P.W.J., STEWART, I.D., MORTENSEN, N.J.McC. & WILLIAMSON, R.C.N. (1986). Programme for early detection of gastric cancer. *Br. Med. J.*, **293**, 883 (Letter).

KELSEN, D.P., BUCKNER, J., EINZEG, A., MAGILL, G., HEELAN, R. & VINCIGUERRA, V. (1987). Phase II trial of cisplatin and etoposide in adenocarcinomas of the upper gastrointestinal tract. *Cancer Treat. Rep.*, **71**, 329.

KLEIN, H.O., DIAS WICKRAMANAYAKE, P., DIETERLE, F., MOHR, R., OERKERMANN, H. & GROSS, R. (1983). High-dose MTX/5-FU and adriamycin for gastric cancer. *Semin. Oncol 1983.*, **10**, 29.

KROOK, J.E., O'CONNELL, M.J. & WIEAND, H.S. (1988). Surgical adjuvant therapy of gastric cancer with doxorubicin and 5-fluorouracil. A joint Mayo Clinic/North Central Cancer Treatment Group study. *Proc. Am. Soc. Clin. Oncol.*, **7**, 93 (*abstract*).

LACAVE, A.J., WILS, J., DIAZ-RUBIO, E. & 5 others (1985). Cisplatin as second-line chemotherapy in advanced gastric adenocarcinoma. A phase II study of the EORTC Gastrointestinal Tract Cancer Cooperative Group. *Eur. J. Cancer Clin. Oncol.*, **21**, 1321.

LACAVE, A.J., WILS, J., DIAZ-RUBIO, E. & 5 others (1985). Cisplatin as second-line chemotherapy in advanced gastric adenocarcinoma. A phase 2 study of the EORTC Gastrointestinal Tract Cancer Cooperative Group. *Eur. J. Cancer Clin. Oncol.*, **21**, 1321.

LACAVE, A.J., ANTON-APARICIO, L., GONZALEZ-BARON, M. & 4 others (1987). Cisplatin (CDDP) and 5-fluorouracil (5FU) 120-hr infusion for advanced gastric cancer (GC): a phase II multicenter study. *Proc. Am. Soc. Clin. Oncol.*, **6**, 91 (*abstact*).

LAVIN, P.T., BRUCKNER, H.W. & PLAXE, S.C. (1982). Studies in prognostic factors relating to chemotherapy for advanced gastric cancer, **50**, 2016.

LEICHMAN, L., McDONALD, B., DINDOGRU, A., SAMPSON, M. & VAITKEVICIUS, V.K. (1984). Cisplatin: an active drug in the treatment of disseminated cancer. *Cancer*, **53**, 18.

LEICHMAN, L., McDONALD, B., DINDOGRU, A., SAMPSON, M. & VAITKEVICIUS, V.K. (1984). Cisplatin: an active drug in the treatment of disseminated cancer. *Cancer*, **53**, 18.

LEVI, J.A., DALLEY, D.N. & ARONEY, R.S. (1979). Improved combination chemotherapy in advanced gastric cancer. *Br. Med. J.*, **2**, 1471.

LEVI, J.A., FOX, R.M., TATTERSALL, M.H., WOODS, R.L., THOMSON, D. & GILL, G. (1986). Analysis of a prospectively randomised comparison of doxorubicin versus 5-fluorouracil, doxorubicin and BCNU in advanced gastric cancer: implications for future studies, *J. Clin. Oncol.*, **4**, 1348.

LONGMIRE, W.P., KIRZMA, J.W. & DIXON, W.J. (1968). The use of triethylene-thiophosphoramide as an adjuvent to the surgical treatment of gastric carcinoma. *Ann. Surg.*, **167**, 293.

MACDONALD, J.S., WOOLLEY, P.V., SMYTHE, T., UENO, W., HOTH, D. & SCHEIN, P. (1979). 5-fluorouracil, adriamycin and mitomycin-C (FAM) combination chemotherapy in the treatment of advanced gastric cancer. *Cancer*, **44**, 42.

MACDONALD, J.S., SCHEIN, P.S., WOOLLEY, M.D. & 8 others (1980). 5-fluorouracil, doxorubicin and mitomycin (FAM) combination chemotherapy for advanced gastric cancer. *Ann. Intern. Med.*, **93**, 533.

MARUYAMA, K. (1986). Results of surgery correlated with staging. In *Cancer of the Stomach*, Preece *et al.* (eds) p. 145. Grune and Stratton: London.

MOERTEL, C.G., MITTLEMAN, J.A., BAKEMEIR, R.F., ENGSTROM, P. & HANLEY, J. (1976). Sequential and combination chemotherapy of advanced gastric cancer. *Cancer*, **38**, 678.

NAKAJIMA, T., HARASHIMA, S., HIRATA, M. & KAJITANI, T. (1978). Prognostic and therapeutic values of peritoneal cytology in gastric cancer. *Acta Cytologica*, **22**, 225.

O'CONNELL, M.J., SCHUTT, A.J., MOERTEL, C.G. & HAHN, R.G. (1987). Phase II clinical trial of trianite in combination with mitomycin-C for patients with advanced gastric cancer. *J. Clin. Oncol.*, **5**, 83.

OFFICE OF POPULATION CENSUSES AND SURVEYS (1986). *Cancer Statistics 1983*. HMSO: London.

PREUSSER, P., WILKE, H., ACHTERRATH, W. & 4 others (1986). Advanced inoperable stomach cancer: a pilot study with the combination etoposide, adriamycin and cisplatin. *Anticancer Res.*, **6,** 1195.

PREUSSER, P., WILKE, H., ACHTERRATH, W. & 4 others (1987). Advanced gastric carcinoma: a phase II study with etoposide (E), adriamycin (A) and split course cisplatin (P) = EAP. *Proc. Am. Soc. Clin. Oncol.*, **6,** 75 (*abstract*).

PREUSSER, P., FINK, U., GUNZER, U. *et al.* (1988). Nonadjuvant chemotherapy with etoposide/adriamycin.cisplatin (EAP) in locally advanced gastric cancer. *Proc. Am. Soc. Clin. Oncol.*, **7,** 100 (*abstract*).

SERLIN, O., WOLKOFF, J.S., AMADEO, J.M. & KEEHN, R.J. (1969). Use of 5-fluorodeoxyuridine (FUDR) as an adjuvant to the surgical management of carcinomas of the stomach. *Cancer*, **24,** 223.

SWYNNERTON, B.F. & TRUELOVE, S.C. (1952). Carcinoma of the stomach. *Br. Med. J.*, **1,** 287.

THE GASTROINTESTINAL TUMOUR STUDY GROUP (GTSG) (1979). Phase II–III chemotherapy studies in advanced gastric cancer. *Cancer Treat. Rep.*, 1871.

THE GASTROINTESTINAL TUMOUR STUDY GROUP (GTSG) (1982). A comparative clinical assessment of combination chemotherapy in the management of advanced gastric cancer. *Cancer*, **49,** 1362.

THE GASTROINTESTINAL TUMOUR STUDY GROUP (GTSG) (1984). Randomised study of combination chemotherapy in unresectable gastric cancer. *Cancer*, **53,** 13.

WAGENER, D.J., YAP, S.H., WOBBES, T. & 6 others (1985). Phase II trial of 5-fluorouracil, adriamycin and cisplatin (FAP) in advanced gastric cancer. *Cancer Chemother. Pharmacol.*, **15,** 86.

WILS, J., BLEIBERG, H., DALESIO, O. & 5 others (1986). An EORTC Gastrointestinal Group Evaluation of the combination of sequential methotrexate and 5-fluorouracil, combined with adriamycin in advanced measureable gastric cancer. *J. Clin. Oncol.*, **4,** 1799.

WILS, J., COOMBES, R.C., CHILVERS, C. & 9 others (1988). Randomized trial of FAM (5-fluorouracil, adriamycin, mitomycin-C) chemotherapy versus control as adjuvant treatment of resected gastric cancer: 4, 5 year results. *Proc. Am. Soc. Clin. Oncol.*, **7,** 97 (*abstract*).